# In-Hospital Obstetric Delays in Rural Uganda: A Cross-Sectional Analysis of a Hospital Cohort

**DOI:** 10.1007/s00268-023-06964-z

**Published:** 2023-03-10

**Authors:** McKayla Poppens, Rasheedat Oke, Melissa Carvalho, Yeranui Ledesma, Silas Okullu, Mary Goretty Ariokot, Esther Agwang, Peter Ekuchu, Hyginus Wange, Marissa Boeck, Catherine Juillard, Mary Margaret Ajiko, Rochelle Dicker

**Affiliations:** 1 David Geffen School of Medicine, University of California Los Angeles, Los Angeles, CA USA; 2 Program for the Advancement of Surgical Equity, Department of Surgery, University of California Los Angeles, Los Angeles, CA USA; 3 Department of Surgery, University of California San Francisco, San Francisco, CA USA; 4 Department of Surgery, Soroti Regional Referral Hospital, Soroti, Uganda

## Abstract

**Background:**

Deaths related to pregnancy and childbirth are extremely high in low-resource countries such as Uganda. Maternal mortality in low- and middle-income countries is related to delays in seeking, reaching, and receiving adequate health care. This study aimed to investigate the in-hospital delays to surgical care for women in labor arriving to Soroti Regional Referral Hospital (SRRH).

**Methods:**

From January 2017 to August 2020, we collected data on obstetric surgical patients in labor using a locally developed, context-specific obstetrics surgical registry. Data regarding patient demographics, clinical and operative characteristics, as well as delays in care and outcomes were documented. Descriptive and multivariate statistical analyses were conducted.

**Results:**

A total of 3189 patients were treated during our study period. Median age was 23 years, most gestations were at term (97%) at the time of operation, and nearly all patients underwent Cesarean Section (98.8%). Notably, 61.7% of patients experienced at least one delay in their surgical care at SRRH. Lack of surgical space was the greatest contributor to delay (59.9%), followed by lack of supplies or personnel. The significant independent predictors of delayed care were having a prenatal acquired infection (AOR 1.73, 95% CI 1.43–2.09) and length of symptoms less than 12 h (AOR 0.32, 95% CI 0.26–0.39) or greater than 24 h (AOR 2.61, 95% CI 2.18–3.12).

**Conclusion:**

In rural Uganda, there is a significant need for financial investment and commitment of resources to expand surgical infrastructure and improve care for mothers and neonates.

## Background

In Sub-Saharan Africa, maternal mortality remains alarmingly high. As of 2017, maternal mortality was 375 per 100,000 live births, accounting for two-thirds of the global burden of maternal deaths [1]. In Uganda, a low-resource country in Sub-Saharan Africa, the situation is no different. The massive difference between the maternal-mortality-ratio of rich and poor countries is one the largest public health disparities [2]. Estimates suggest at least 60% of maternal mortality is attributable to five conditions occurring around labor and the 24-h postpartum period: postpartum hemorrhage, puerperal sepsis, pre-eclampsia or eclampsia, obstructed or prolonged labor, and abortion complications [3].

Access to perinatal care is crucial to minimize maternal and neonatal deaths, however many delays to reaching and receiving care exist. The “Three Delays” model proposes maternal mortality in LMICs is related to delays in: (1) seeking appropriate medical care when an obstetric emergency arises, (2) reaching an obstetrics facility, and (3) receiving appropriate care once reaching a care center [4]. The first delay, seeking timely care, may be related to sociocultural factors, clinician shortage, and financial constraints, among others. The second delay ensues when travel to an appropriate facility is extensively delayed. The last delay occurs in the health facility, and evidence emphasizes that reducing this delay may have the greatest impact on maternal outcomes [5].

In-hospital barriers to maternal healthcare in the developing country context are most often cited due to inadequate training/skills, followed by drug procurement/logistics problems, staff shortages, lack of equipment, and low staff motivation [6]. Studies assessing the relative impact of the three phases of delay contributing to maternal mortality in Ethiopia, Nigeria, Malawi, Indonesia, and Zimbabwe cited in-hospital delays more than either delays in seeking or reaching appropriate medical care [6].

The precise factors contributing to high maternal and neonatal mortality in Uganda are unknown. This study was undertaken to better understand these drivers. Through a collaboration between Ugandan and American partners, a prospective registry was developed at a regional referral hospital in eastern Uganda to collate comprehensive obstetric surgical data beginning in 2017. This study aimed to investigate the third delay to surgical care for women in labor arriving to Soroti Regional Referral Hospital (SRRH).

## Methods

### Study setting

We conducted a cross-sectional survey between January 2017 and August 2020 at SRRH, one of 13 regional hospitals in Uganda. SRRH offers the second-highest level of care in the country, having a referral base of eight district hospitals in the region and serving 21,000 inpatients and 103,000 outpatients annually. This 250-bed, government-run facility serves a rural catchment of 5% of the Ugandan population—about two million people [7]. The obstetrics and gynecology unit is served by a single theater with two operating tables staffed by two obstetricians and a trained anesthetist. Pregnant mothers are evaluated upon arrival in the general outpatient clinic and admitted if necessary.

### Data collection

A structured, 2-page obstetrics surgical registry questionnaire (Appendix 1) was developed based on a literature review and discussions with hospital doctors. Trained research assistants completed the questionnaire during the patient encounter and from medical records to collect data on demographics, prenatal care, preliminary clinical assessment and vital signs, operations, interventions, and outcomes. Data were transferred to an electronic, secure, web-based, REDCap database hosted on the University of California, San Francisco and Los Angeles servers [8]. Inclusion criteria for the registry were patients receiving a surgical operation for an obstetric condition. Delays in surgery were defined by the managing team when surgery was recommended by a surgeon, but lack of necessary factor(s) such as surgical space, personnel, supplies, or other concerns delayed the operation. The managing team evaluated times of arrival, decision for operation, and time of surgery to determine if the patient had in-hospital delays based upon the surgery and indication for surgery. Providers reported the presence of delay and contributing factor, which were then captured in the registry by the trained research assistants. For elective surgery, delay was defined when patients were admitted for operation but faced unforeseen delay due to surgical space, personnel, or supply limitations at the time of surgery preparation.

### Data analysis

Patients were divided into two study populations based upon report of in-hospital delay. Descriptive statistics and tabulations were utilized for demographic, prehospital care, clinical factors, operation, and surgical delay. Chi-squared tests and Fisher’s exact tests were used to identify associations between epidemiological, clinical, and operative characteristics and delays of care. For age, gravida, para, and abortus variables, a Wilcoxon Rank Sum Test was used. A multivariate logistic regression model was built to identify significant factors associated with delay. Variables included in the delays model were those with statistical significance on bivariate analysis. The statistical significance threshold for our analyses was an alpha value of 0.05. Analyses were performed using Stata, version 16.1[9]. Survey entries with missing or duplicate survey data were excluded from the study (*n* = 75). An additional 192 patients not in labor were captured in the registry and also excluded from this study.

### Ethical approval

Verbal informed consent was obtained for all adult patients during their hospital encounter, with permission from guardians obtained for all patients under 18 years of age. Patients under the age of 18 gave assent to the permission granted by their guardians for participation. The study protocol was approved by the SRRH administration and the Institutional Review Board of the University of California, Los Angeles.

## Results

### Demographic and clinical characteristics

In the 44-month study, 3189 patients in labor were captured in the non-trauma obstetrics surgical registry. The median age for all women in labor was 23 (Interquartile range (IQR) 19–28) years. Median obstetric history was gravida 2 (IQR 1–4), para 1 (IQR 0–2), abortus 0 (IQR 0–0), and nearly all patients’ pregnancies (97%) were at term. Of all patients, 1969 (61.7%) experienced at least one delay in receiving their surgical care. (Table 1).

**Table 1:** Demographics and operative characteristics of obstetric surgical patients at SRRH (n = 3189)

Characteristic	All patients *n* = 3189 *n* (%)*	No delay *n* = 1220 *n* (%)*	Delay *n* = 1969 *n* (%)*	*p*-value
Age, median (IQR)	23 (19, 28)	23 (19, 29)	23 (19, 28)	0.26
Gravidity, median (IQR)	2 (1, 4)	2 (1, 4)	2 (1, 4)	0.20
Parity, median (IQR)	1 (0, 2)	1 (0, 3)	0 (0, 2)	0.13
Abortus, median (IQR)	0 (0, 0)	0 (0, 0)	0 (0, 0)	0.78
Gestational age at term	3092 (97.0)	1178 (96.6)	1914 (97.2)	0.30
Comorbidities	106 (3.3)	39 (3.2)	67 (3.4)	0.75
Pregnancy induced condition	92 (2.9)	39 (3.2)	53 (2.7)	0.41
Pregnancy acquired infection	2501 (78.4)	854 (70.0)	1647 (83.6)	< 0.01**
Referred from another hospital	1774 (55.6)	663 (54.3)	1111 (56.4)	0.25
Length of symptoms				< 0.01**
< 12 h	640 (20.1)	448 (36.7)	192 (9.8)	
13 – 24 h	1196 (37.5)	479 (39.3)	717 (36.4)	
> 24 h	1325 (41.5)	280 (23.0)	1045 (53.1)	
Reason for operation		0.03**		
Abnormal lie	173 (5.4)	61 (5.0)	112 (5.7)	
Amniotic fluid disorder	73 (2.3)	30 (2.5)	43 (2.2)	
Cephalopelvic disproportion	400 (12.5)	132 (10.8)	268 (13.6)	
Elective Cesarean section	57 (1.8)	31 (2.5)	26 (1.3)	
Fetal distress	297 (9.3)	117 (9.6)	180 (9.1)	
Hemorrhage	99 (3.1)	38 (9.6)	61 (3.1)	
Hypertensive disorder	61 (1.9)	21 (1.7)	40 (2.0)	
Intrauterine fetal death	20 (< 0.01)	8 (< 1)	12 (< 1)	
Maternal comorbidities	93 (2.9)	43 (3.5)	50 (2.5)	
Multifetal pregnancy	70 (2.2)	21 (1.7)	49 (2.0)	
Placenta previa	54 (1.7)	27 (2.2)	27 (1.4)	
Premature membrane rupture	41 (1.3)	20 (1.6)	21 (1.1)	
Previous Cesarean section	671 (21.0)	239 (19.6)	432 (2.2)	
Prolonged or obstructed labor	1073 (33.6)	430 (35.2)	643 (3.3)	
Unknown	7 (< 1)	2 (< 1)	5 (< 1)	
Emergency surgery	3107 (97.4)	1178 (96.5)	1929 (98.0)	0.01**
*Operation*				
Cesarean section	3152 (98.8)	1199 (98.3)	1953 (99.2)	0.02**
Obstetric ward-to-theater time		< 0.01**		
< 30 min	403 (12.6)	211 (17.3)	192 (9.8)	
31–60 min	2400 (75.3)	807 (66.1)	1593 (80.9)	
61–90 min	76 (2.4)	31 (2.7)	45 (2.3)	
> 90 min	37 (1.2)	4 (< 1)	44 (2.2)	
Unknown	273 (8.6)	167 (1.4)	106 (5.4)	

IQR, Interquartile range

*Frequency and percentages unless otherwise specified

**Indicates *p*-value less than 0.05

Pregnancy acquired infections were reported in 2,501 (78.4%) patients and having at least one infection was significantly associated with surgical delay (*p* < 0.01). Malaria (*n* = 504, 15.8%) and urinary tract infections (UTIs) (*n* = 1991, 62.4%) accounted for nearly all infections.

Length of symptoms varied among patients with 640 (20.1%) reporting symptoms of less than 12 h prior to seeking care and 1325 (41.5%) experiencing symptoms for more than 24 h prior to presentation (*p* < 0.01) (Table 1).

### Operative characteristics

Indications for surgery varied amongst women—prolonged or obstructed labor accounted for 33.6% of operations (*n* = 1073). Reasons for operation were significantly associated with operative delay (*p* = 0.03). Cesarean section accounted for 98.8% (*n* = 3152) of all operations. For 7 cases, the type of surgery was not specified. Surgeries deemed emergent by the obstetrician (*n* = 3107, 97.4%) were associated with delay as compared to nonemergent surgeries (*p* = 0.01) (Table 1).

### Causes of surgical delays

**Fig. 1:**
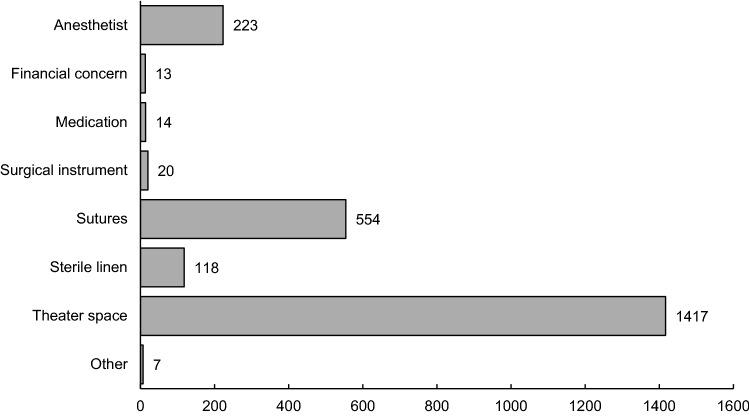
Delays encountered for obstetric surgical patients at SRRH (*n* = 2366 total delays encountered by 1969 patients)*. *375 patients experienced > 1 delay

Most patients (*n* = 1969, 61.7%) experienced at least one delay to their surgery. For 12% of patients, two or more surgical delays were encountered. Lack of theater space was the most frequent limiting factor (1417, 59.9%), followed by lack of sutures (*n* = 554, 23.4%), unavailability of a trained anesthetist (*n* = 223, 9.4%), and lack of sterile linens (*n* = 118, 5.0%). A small percentage of operations were delayed due to lack of other materials: surgical instruments or medications. Other delays (*n* = 7) were due to lack of blood, unavailability of a surgeon, delay in necessary imaging, or unknown reason (Fig. 1).

### Significant predictors of delay

Results of a multivariate logistic regression demonstrated that prenatal acquired infection (AOR 1.73, 95% CI 1.43–2.09), length of symptoms less than 12 h (AOR 0.32, 95% CI 0.26–0.39) and symptoms greater than 24 h (AOR 2.61, 95% CI 2.18–3.12) were independent significant predictors of surgical delay (Table 2).

**Table 2:** Multivariate logistics regression of significant predictors of delays in care for obstetric surgical patients at SRRH

Predictors	COR (95% CI)	*p*-value	AOR (95% CI)	*p*-value
*Prenatal acquired infection*				
Yes	2.19 (1.85 – 2.60)	< 0.01*	1.73 (1.43 – 2.09)	< 0.01*
No	Ref	Ref	Ref	Ref
*Length of symptoms*				
< 12 h	0.18 (0.15 – 0.22)	< 0.01*	0.32 (0.26 – 0.39)	< 0.01*
12–24 h	Ref	Ref	Ref	Ref
> 24 h	3.80 (3.23 – 4.46)	< 0.01*	2.61 (2.18 – 3.12)	< 0.01*
*Reason for operation*				
Abnormal lie	1.15 (0.84 – 1.58)	0.41	1.48 (0.61 – 3.60)	0.40
Amniotic fluid disorder	0.89 (0.56 – 1.42)	0.61	1.07 (0.41 – 2.82)	0.89
Cephalopelvic disproportion	1.30 (1.04 – 1.62)	0.02*	1.51 (0.65 – 3.55)	0.34
Elective Cesarean section	Ref	Ref	Ref	Ref
Fetal distress	0.95 (0.74 – 1.21)	0.67	1.29 (0.54 – 2.74)	0.57
Hemorrhage	0.99 (0.66 – 1.50)	0.98	1.08 (0.43 – 2.74)	0.90
Hypertensive disorder	1.18 (0.69 – 2.02)	0.54	1.20 (0.44 – 3.27)	0.72
Intrauterine fetal death	0.93 (0.38 – 2.30)	0.87	0.68 (0.19 – 2.36)	0.54
Maternal comorbidities	0.72 (0.47 – 1.08)	0.11	0.91 (0.38 – 2.21)	0.84
Multifetal pregnancy	1.46 (0.87 – 2.44)	0.15	1.77 (0.66 – 4.72)	0.25
Placenta previa	0.61 (0.36 – 1.05)	0.08	0.75 (0.27 – 2.04)	0.57
Premature membrane rupture	0.65 (0.35 – 1.20)	0.17	0.94 (0.62 – 3.31)	0.91
Previous Cesarean section	1.15 (0.97 – 1.38)	0.11	1.43 (0.62 – 3.31)	0.41
Prolonged or obstructed labor	0.89 (0.77 – 1.04)	0.13	1.21 (0.53 – 2.80)	0.53
Unknown	1.55 (0.30 – 8.00)	0.60	1.83 (0.18 – 18.5)	0.61
*Type of surgery*				
Emergency surgery	1.82 (1.15 – 2.90)	0.01*	1.53 (0.74 – 3.14)	0.25
Nonemergent surgery	Ref	Ref	Ref	Ref

*Indicates *p*-values less than 0.05

COR, Crude Odds Ratio

AOR, Adjusted Odds Ratio

### Outcomes

Eighty-one patients (2.5%) experienced a surgical complication. Three patients died (cardiac arrest, disseminated intravascular coagulation, and an unspecified death, < 1%), and four patients (< 1%) had a new long-term disability following surgery. No adverse outcome was associated with having experienced a surgical delay.

**Fig. 2:**
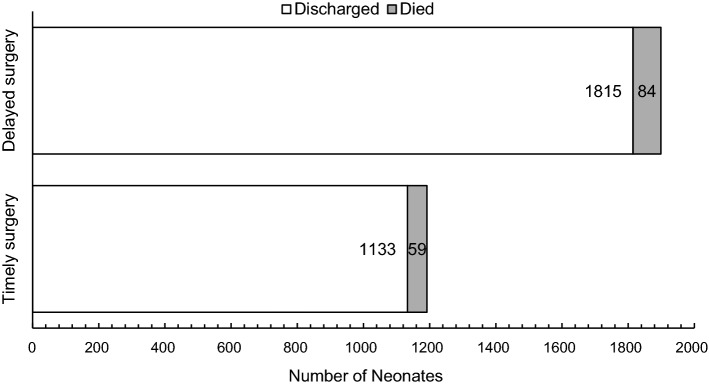
Neonatal mortality for timely and delayed obstetric surgeries at SRRH, *p* = 0.50

Overall, the neonatal in-hospital mortality rate was 46 per 1000 live births (4.6%), with most deaths being stillbirth (*n* = 118). Neonatal mortality was not associated with surgical delay (Fig. 2). Neonatal deaths occurring within 24 h of birth were significantly less likely to be associated with surgical delay (*p* < 0.01). Neonatal mortality was significantly associated with patients who were referred from another care center (*p* < 0.01). The neonatal mortality of referred patients was 58 per 1000 live births (5.8%), as compared to the neonatal mortality of 32 per 1000 live births (3.2%) for patients cared for solely at SRRH (Table 3).

**Table 3:** Obstetric surgical patient and neonate outcomes at SRRH

Maternal outcome	All patients *n* = 3189 *n* (%)	No delay *n* = 1120 *n* (%)	Delay *n* = 1969 *n* (%)	*p*-value
Surgical complication	81 (2.5)	23 (2.1)	58 (2.9)	0.06
Death	3 (< 1)	0 (0)	3 (< 1)	0.29
New Long-term disability	4 (< 1)	1 (< 1)	3 (< 1)	1.00
Neonatal outcome	All Neonates *n* = 3091 *n* (%)	No Delay *n* = 1192 *n* (%)	Delay *n* = 1889 *n* (%)	*p*-value
Neonatal mortality*	143 (4.6)	59 (4.9)	84 (4.4)	0.50
Stillbirth	118 (3.8)	44 (3.6)	74 (3.9)	0.82
Less than 24 h	14 (4.5)	11 (< 1)	3 (< 1)	< 0.01*
24–48 h	7 (< 1)	3 (< 1)	4 (< 1)	0.80
More than 48 h	4 (< 1)	1 (< 1)	3 (< 1)	0.56
Neonatal outcome	All neonates *n* = 3091 *n* (%)	Not referred *n* = 1357 *n* (%)	Referred *n* = 1734 *n* (%)	*p*-value
Neonatal mortality	143 (4.6)	43 (3.2)	100 (5.8)	< 0.01*

*Neonatal mortality measured from time of birth

## Discussion

This study highlights the epidemiological and clinical presentations of women in labor presenting to SRRH and demonstrates important delays in care. The significant independent predictors of delayed care were having a prenatal acquired infection and duration of symptoms. The most common surgical delay was operating theater space, accounting for two-thirds of the delays for obstetric patients. SRRH has one operating theater with two operating tables used for both obstetrical and general surgical operations, meaning if the space is occupied when obstetrical emergencies arise, there is an inherent delay.

The overwhelming number of obstetric emergency cases are compounded by deficits that trouble many hospitals in LMICs [10]. Across the literature, the most frequent barriers of in-hospital delays to maternal healthcare in low-resourced settings are related to human resources [6]. A northern Ugandan study using qualitative interviewing to identify in-hospital delays to receiving emergency cesarean sections across three hospitals and 13 primary healthcare centers found many similar delays to this study: shortage of medicine and supplies, lack of blood and functioning theater space, gaps in staff coverage or skill, and delays in facility referral [11]. These findings support a theme that shortage of supplies and necessary space are major barriers to maternal healthcare across Uganda. Although it is Ugandan governmental policy that all surgical care delivered at government hospitals in Uganda is free of charge, in practice, broken equipment and frequent stock-outs require patients to pay out-of-pocket for large portions of their care, adding financial burden and time to receive appropriate care [12]. The regularity of delays in surgical care highlights a critical shortcoming in the receipt of quality, timely maternal care, especially in the position SRRH serves as a referral hospital.

Patients with acquired infections had nearly twice the odds of having a surgical delay as compared to mothers without infections. Cautionary measures taken during pre-operative care may have contributed to the higher likelihood of having delayed surgeries. Patients with malarial or complicated urinary tract infections were often febrile upon arrival, and in these cases, choice and obtainment of appropriate anesthesia and antibiotics added additional preparatory steps that patients without infections did not face. Malaria in pregnancy is associated with higher rates of miscarriage, intrauterine demise, premature delivery, neonatal low-birth weight, and neonatal death [13]. If untreated, UTIs can cause chorioamnionitis, which is the most common infection-related complication in labor-and-delivery units worldwide [14]. Although this poses significant morbidity risk to a mother and the fetus, no evidence supports that immediate delivery after intrapartum diagnosis prevents adverse maternal and neonatal outcomes, or long-term neurodevelopmental outcomes [14]. Treating maternal infections in the prepartum period may reduce a mother’s odds of having a delayed operation at SRRH, but if treated intrapartum, this is unlikely to prevent adverse outcomes.

Symptom duration also predicted odds of surgical delay. Patients presenting to SRRH within 12 h of symptom onset had a lower odds of delay, while those reporting symptoms for more than 24 h had a higher odds of delayed operation. This suggests patients presenting acutely may have presented in greater distress, prompting operation prioritization. Symptom specification upon presentation would improve our understanding of this association in the future.

Though not statistically significant contributors, operation indication and acuity were related to delay. Elective and non-emergent operations had fewer surgical delays. In these cases, adequate time to staff and stock the operative theaters was likely responsible for the lower probability of delay.

Despite the abundance of surgical delays, they were not statistically significant contributors to maternal or neonatal mortality. In fact, timely surgery did not guarantee neonatal safety. Mothers with obvious distress or worse presentation may have received surgery sooner but had worse neonatal outcomes. In this study, referral from an outside facility is a marker of surgical acuity as patients requiring higher level of care are referred to SRRH. Higher rates of neonatal mortality among referred cases suggests the acuity of an obstetric emergency impacted neonatal outcome more than encountered delays. The difference in outcomes associated with intervention timeliness may be obscured by this selection bias. Nonetheless, other studies have shown that failure to achieve the suggested decision-to-incision time of under 30 min does not negatively impact neonatal outcome or maternal complications [15, 16]. Neonatal mortality, however, may increase significantly if Cesarean section is delayed by over 2 hours [17].

### Limitations

This study is not without limitations, predominantly related to the nature of the survey data used. Analyses largely relied on women’s recall of details about prenatal care, comorbidities, and pregnancy-included conditions. Reliance on self-recall imparts possible reporting-bias, although the extent of recall-bias has not been assessed in Uganda. Surgical complications were captured from surgical notes, so incomplete surgical notes and limited medical charting may have led to inaccurate record of surgical complications, a similar finding to that of the SRRH trauma surgery registry [18]. Precise time of arrival, time to decision, and time to surgery were not recorded. Surgical urgency was determined by the obstetrician, and thus, surgical delay was a subjective measure. Lastly, patient follow-up was difficult post-operation. Our study’s death rate was less than 0.1%, lower than the national average maternal mortality of 0.375% [1]. We recognize this study’s maternal mortality rate may not represent true hospital maternal mortality since staff shortage made capturing patient outcomes difficult.

## Conclusion

This study sheds light on the in-hospital delays that obstetric surgical patients experience in a rural referral hospital in Uganda. Obstetric patients at SRRH endure significant delays in their surgical care, primarily due to infrastructure deficits. Improvements in pre-hospital care to reduce the length of symptoms and the referral process from other facilities may also improve in-hospital delays and neonatal outcomes. Infrastructure and resource investments could improve care for the thousands of mothers receiving surgical interventions at SRRH annually. Future directions will focus on implementation of a revised obstetrics surgical registry questionnaire and assessment of quality improvement measures as they pertain to the delays identified in this study.
